# The Relationship between Epigenetic Age and Myocardial Infarction/Acute Coronary Syndrome in a Population-Based Nested Case-Control Study

**DOI:** 10.3390/jpm12010110

**Published:** 2022-01-14

**Authors:** Sofia Malyutina, Olga Chervova, Taavi Tillmann, Vladimir Maximov, Andrew Ryabikov, Valery Gafarov, Jaroslav A. Hubacek, Hynek Pikhart, Stephan Beck, Martin Bobak

**Affiliations:** 1 Research Institute of Internal and Preventive Medicine-Branch of Institute of Cytology and Genetics SB RAS, 630089 Novosibirsk, Russia; medik11@mail.ru (V.M.); a_ryabikov@hotmail.com (A.R.); valery.gafarov@gmail.com (V.G.); 2 UCL Cancer Institute, University College London, London WC1E 6BT, UK; o.chervova@ucl.ac.uk (O.C.); s.beck@ucl.ac.uk (S.B.); 3 Institute for Global Health, University College London, London WC1E 6BT, UK; t.tillmann@ucl.ac.uk; 4 Experimental Medicine Centre, Institute for Clinical and Experimental Medicine, 14021 Prague, Czech Republic; jahb@ikem.cz; 5 Institute of Epidemiology and Health Care, University College London, London WC1E 6BT, UK; h.pikhart@ucl.ac.uk (H.P.); m.bobak@ucl.ac.uk (M.B.)

**Keywords:** DNA methylation, epigenetic age, myocardial infarction, acute coronary syndrome, population, nested case-control, HAPIEE project

## Abstract

We investigated the relationship between ‘epigenetic age’ (EA) derived from DNA methylation (DNAm) and myocardial infarction (MI)/acute coronary syndrome (ACS). A random population sample was examined in 2003/2005 (*n* = 9360, 45–69, the HAPIEE project) and followed up for 15 years. From this cohort, incident MI/ACS (cases, *n* = 129) and age- and sex-stratified controls (*n* = 177) were selected for a nested case-control study. Baseline EA (Horvath’s, Hannum’s, PhenoAge, Skin and Blood) and the differences between EA and chronological age (CA) were calculated (ΔAHr, ΔAHn, ΔAPh, ΔASB). EAs by Horvath’s, Hannum’s and Skin and Blood were close to CA (median absolute difference, MAD, of 1.08, –1.91 and –2.03 years); PhenoAge had MAD of −9.29 years vs. CA. The adjusted odds ratios (ORs) of MI/ACS per 1–year increments of ΔAHr, ΔAHn, ΔASB and ΔAPh were 1.01 (95% CI 0.95–1.07), 1.01 (95% CI 0.95–1.08), 1.02 (95% CI 0.97–1.06) and 1.01 (0.93–1.09), respectively. When classified into tertiles, only the highest tertile of ΔAPh showed a suggestion of increased risk of MI/ACS with OR 2.09 (1.11–3.94) independent of age and 1.84 (0.99–3.52) in the age- and sex-adjusted model. Metabolic modulation may be the likely mechanism of this association. In conclusion, this case-control study nested in a prospective population-based cohort did not find strong associations between accelerated epigenetic age markers and risk of MI/ACS. Larger cohort studies are needed to re-examine this important research question.

## 1. Introduction

Increasing life expectancy worldwide is accompanied by an aging population. Currently, there are at least 900 million people over 60 years old in the world and, according to the United Nations estimates, the world’s population is expected to reach 8.6 billion people by 2030, of which more than 1.4 billion will be over the age of 60 [1].

Cardiovascular diseases (CVDs), particularly atherosclerotic CVDs such as coronary heart disease (CHD) and cerebrovascular diseases, are the leading cause of mortality and morbidity, being responsible for about 30% of global deaths [2], and aging is a major risk factor. In the multidimensional process of decline in health status during aging, molecular markers of ‘biological age’ are regarded as determinants of the rate of aging. Epigenetic modifications such as DNA methylation (DNAm) have been shown to be the most accurate molecular readout of aging but their possible functional role remains poorly understood [3,4]. Altered epigenetic patterns may therefore be important causes and/or signals of this aging process.

Epigenetic modifications are mitotically (and in some cases meiotically) heritable. They can change gene and genome function independently of changes in the nucleotide sequence of the DNA [5]. The epigenetic phenomenon of DNAm involves the addition or removal of a methyl group to the 5’ cytosines, most commonly in the context of CpG dinucleotides; areas of relatively high CpG density are referred to as CpG islands which are often associated with a gene promoter [6]. DNAm can be measured quantitatively, and it is increasingly used in human studies [7].

It has been repeatedly shown that DNAm levels at specific sites in the genome are strongly associated with age and that, in some cases, it has been used to accurately predict chronological age [6,8,9,10]. These sites underline the concept of ‘epigenetic clocks’ and several DNAm-based estimators of chronological age, referred to as ‘epigenetic age’, have been constructed [11]. Hannum’s Blood-based clock is based on 71 CpG sites [9], Horvath’s Pan-Tissue clock is based on 353 CpG sites [12], Levine’s PhenoAge clock is based on 513 CpG sites capturing age-related and functional phenotype modifications [13] and Horvath’s Skin and Blood clock is based on 391 CpGs for human fibroblasts and other cell types [14]. Further to these, over 30 epigenetic clocks have been published [15], including those recently developed on the base of the Illumina Methylation EPIC 850 BeadChip (850 K) [16].

In our study, we chose to use Horvath’s and Hannum’s clocks because they are among the most popular first-generation clocks and are featured (and continue to be used) in many studies of associations between age and phenotypes. The Skin and Blood clock was chosen as an example of a specialized second-generation clock which is known in the research community as the most accurate chronological age predictor reported to date. PhenoAge, which is another second-generation clock, was also chosen based on its popularity among age–trait association studies, including mortality.

We would like to note that second-generation clocks (PhenoAge, GrimAge [17], etc.) are less precise in terms of chronological age prediction in comparison to first-generation or specialized epigenetic clocks. This is because those clocks were designed to incorporate other phenotypes (or comorbidities) and were not primarily aimed at reflecting chronological age. In our experience, PhenoAge is usually well below the chronological age in the vast majority of moderately healthy individuals, which is supported by several publications, including [13,18].

A number of studies have shown an association between epigenetic age and risk of mortality, as summarized in various meta-analyses [19,20,21,22,23,24]. Both positive and negative correlations have been made on the relationship between epigenetic age and CVD and, specifically, CHD [15,21,24,25], however, these studies are limited, heterogeneous in design and the findings remain largely inconclusive.

In Russia, the proportion of the elderly population is growing, but life expectancy at birth remains on average 8 years lower than in Western Europe [26,27], although the causes of this gap remain unclear [28,29,30]. This points toward the need to understand all aspects of aging in the Russian population. There have been no longitudinal studies of the relationship between epigenetic measures of age and CVD and chronic diseases in the Russian population. For these reasons, the current analysis is relevant both locally and across the world at large.

Objective of our study was to investigate the relationship between epigenetic age (EA) and myocardial infarction (MI)/acute coronary syndrome (ACS) in a population-based nested case-control study.

## 2. Methods

### 2.1. Study Population and Design

A random population sample was examined in the Russian arm of the HAPIEE study at baseline in 2003/05 (*n* = 9360, age 45–69) and re-examined in 2006–2008 and 2015–2017 The cohort was followed up until 31.12.2019 for an average of 15.9 (SD 0.64, median 15.9) years for fatal and non-fatal cardiovascular events and all-cause mortality.

Data on fatal and non-fatal coronary heart disease (CHD) (ICD–10: I20–I25) events were collected at the Research Institute of Internal and Preventive Medicine (IIPM) using a Register of Myocardial Infarction originally established in the WHO MONICA project by combining ‘hot pursuit’ and ‘cold pursuit’ methods and using medical records and hospital discharge reports. The data on all-cause and cause-specific mortality were collected at the IIPM using various sources, including the Population Registration Bureau (ZAGS) and the Novosibirsk Office of the State Statistical Bureau (Rosstat), and information received at repeated waves of the study (this includes the address bureau, as well as contacts with relatives of deceased study participants).

### 2.2. Sample Selection Process

During a 15-year follow-up period, 1475 events of myocardial infarction (MI) or acute coronary syndrome (ACS) were ascertained in 9360 unique persons including serial events in some individuals. Using a nested case-control study design, we applied the following exclusion criteria for selecting MI/ACS cases in this study: prevalent baseline CVD (MI, ACS, stroke), data not available for DNA analysis. Exclusion criteria for selection in the control group of this study were the same, with additional exclusion of controls with baseline cancer or those who died before the end of follow-up. We then randomly selected participants with incident MI/ACS (cases) and age- and sex-frequency matched controls. Assuming that a small proportion of DNA samples would be unavailable or rejected by quality control, we selected initially 161 cases and 243 controls. Among them, 139 cases and 187 controls were available and appropriate for DNAm profiling. After DNAm quality control (procedures are described below), 129 cases and 177 controls were included in the analysis. DNAm profiles of 88 subjects available from the earlier pilot study [31] were included in the sampling algorithm and were included in the ‘expanded control group’ (*n* = 265) for additional analyses. The general characteristics of expanded controls are summarized in Table S2.

### 2.3. Ethics

All study participants provided informed consent and study protocols were approved by the ethical committee of the Research Institute of Internal and Preventive Medicine. The study was conducted in accordance with the relevant ethical guidelines and regulations.

### 2.4. Data Collection

Baseline data collection in the HAPIEE study was conducted using a comprehensive questionnaire, medical examination and the collection of venous blood samples. The protocol included assessment of history of cardiovascular and other chronic diseases, lifestyle habits and health, socio-economic circumstances, objective measurement of blood pressure (BP), anthropometric parameters and physical performance. The details of the protocol are reported elsewhere [32].

The lifestyle habits, health and socio-economic circumstances were assessed by structured interview. A person who smoked at least one cigarette a day was classified as a smoker. Smoking status was categorized as current smoker, former smoker and never smoked.

The level of education was categorized into 4 categories (high, secondary, vocational and primary or less than primary education). For the current analysis, marital status was dichotomized as married (or cohabiting) and single (never been married, divorced or widower/widow).

The height and weight were measured with accuracy to 1 mm and 100 g, respectively; body mass index (BMI) was calculated as kg/m^2^. Blood pressure (BP) was measured three times (Omron M-5 tonometer) on the right arm in a sitting position after a 5 min rest period with a 2 min interval between measurements. The average of three BP measurements was calculated.

Blood samples were drawn following at least 8 h of fasting. Serum was stored at minus 80 °C and analyses for lipids and glucose were conducted within one month after sample collection. The levels of total cholesterol (TC), triglycerides (TG), high-density lipoprotein cholesterol (HDLC) and glucose in blood serum were measured enzymatically by a KoneLab Prime 30i autoanalyzer (Thermo Fisher Scientific Inc., Waltham, MA, USA) using kits from Thermo Fisher Scientific (Thermo Fisher Scientific Inc., Waltham, MA, USA). Low-density lipoprotein cholesterol (LDLC) was calculated using the Friedewald formula.

Genomic DNA was isolated from whole blood cells by phenol-chloroform extraction and stored at minus 70 °C until further laboratory analysis.

### 2.5. DNAm Profiling

Whole blood DNAm profiling was performed using Illumina Infinium Methylation EPIC BeadChip arrays following the manufacturer’s recommended protocol (Illumina Inc, San Diego, CA, USA). The arrays were scanned using the iScan Microarray Scanner with an autoloader (Illumina Inc, San Diego, CA, USA) to produce the raw signal intensity files (.idat files) in accordance with standard operating procedures.

### 2.6. Data Preprocessing and Quality Control (QC)

All the data preprocessing and QC procedures were performed using R version 4.1.0 (R Foundation for Statistical Computing, Vienna, Austria) and dedicated R libraries minfi [33], ChAMP [34] and ENmix [35], following the steps described in [36]. In particular, our QC checks included array control probes’ metrics as described in Illumina Bead Control Reporter guidelines, detection *p*-values and bead count numbers. In addition, we inspected the concordance of the reported sex with one inferred from DNAm data, and for the samples with available repeated DNAm profiling at a different time point, we performed sample matching using the data from 59 EPIC array SNP control probes. In our analysis, we only used data from the samples with less than 1% CpGs with detection *p*-values above the threshold 0.01, and probes (CpGs) with bead count numbers of at least 3 and *p*-values below 0.01 across at least 99% of samples.

### 2.7. DNAm Age Calculation

Baseline EA was calculated using Horvath’s [12], Hannum’s [9], PhenoAge [13] and Skin and Blood DNAm clocks [14]. The missing probes required for the DNAm age calculation were imputed using the kNN method [37,38], implemented in the ENmix R library. Following the definition in [12], we calculated age acceleration as a difference between EA and chronological age (CA) for each clock. Corresponding age accelerations for Horvath’s, Hannum’s, PhenoAge and Skin and Blood clocks were denoted as ΔAHr, ΔAHn, ΔAPh and ΔASB, respectively.

### 2.8. Statistical Analysis 

Statistical analysis was conducted using SPSS (v19.0, Inc., Chicago, IL, USA) and R (v4.1.0) software packages (R Foundation for Statistical Computing, Vienna, Austria). The dataset includes 129 MI/ACS cases and 177 controls.

First, descriptive analysis compared chronological age (CA), EA and general characteristics of case and control groups using ANOVA and cross-tabulation techniques.

Second, logistic regression was used to estimate odds ratios of MI/ACS per 1–year increment of EA as a continuous variable. The dependent variable was cases of incident MI/ACS. Model 1 was adjusted for baseline age; Model 2 was adjusted for age and sex; Model 3a was adjusted for age, sex and smoking; Model 4 was adjusted for age, sex, smoking, systolic blood pressure (SBP), total cholesterol (TC), body mass index (BMI) and education level.

Finally, we classified subjects into tertiles of the difference between EA and CA for the four EA measures (ΔAHr, ΔAHn, ΔAPh, ΔASB), and logistic regression was used to estimate odds ratios of MI/ACS by EA tertile using cases of incident MI/ACS as the dependent variable. For the independent variable (difference between EA and CA), the reference category consisted of the tertile of participants with the smallest EA–CA difference. The tertile cutpoints were ΔAHr (−1.38; 3.26), ΔAHn (−3.95; 0.13), ΔAPh (−11.50; −6.21), ΔASB (−3.52; −0.31). Age-adjusted and multivariable-adjusted models were estimated with the same covariates as above.

## 3. Results

### 3.1. Cases and Controls Have Significant Differences in Basic Phenotype Characteristics

After the quality control procedures, the analytical sample consisted of 129 cases and 177 controls. The general characteristics of case and control participants are summarized in Table 1.

The individuals with incident cases of MI/ACS were slightly older, as expected, they had higher BP, anthropometric measures (body mass index, BMI, and waist/hip ratio, WHR) and levels of plasma glucose, more frequently had hypertension (HT) and type 2 diabetes (DM2) and were less educated compared to controls.

**Figure 1:**
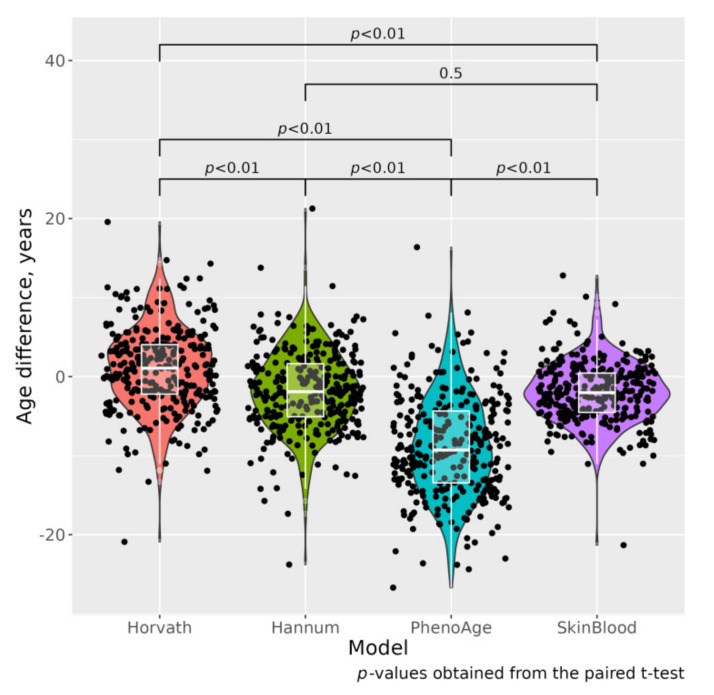
Boxplots of differences between chronological and epigenetic age (cases and controls, *n* = 306).

**Figure 2:**
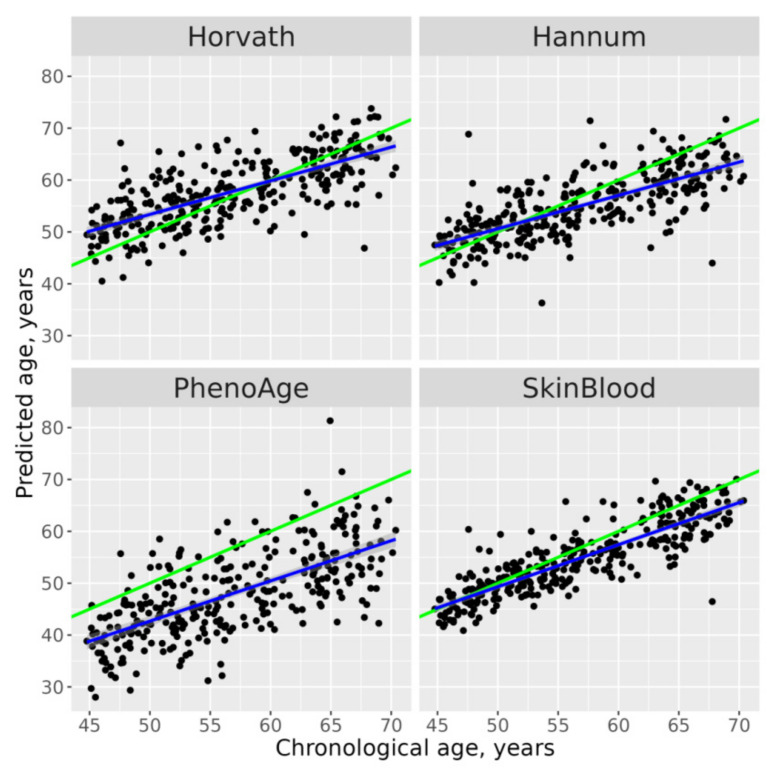
Scatterplots of chronological vs. epigenetic age by Horvath’s, Hannum’s, PhenoAge and Skin and Blood clocks. Diagonal green line corresponds to the predicted age equal to the chronological age, blue straight line corresponds to the linear regression.

DNAm ages calculated by Horvath’s, Hannum’s and Skin and Blood clocks were similar to participants’ CA; the corresponding median absolute differences (MADs) were 1.08, −1.91 and −2.03 years (Figure 1). Means (SD) were 0.98 (5.25), −1.81 (5.10) and −1.97 (3.81) for ΔAHr, ΔAHn and ΔASB, respectively. As expected, PhenoAge’s predictions were less precise with MAD = −9.29 and ΔAPh mean (SD) −8.84 (6.39). Scatterplots of chronological vs. epigenetic age by Horvath’s, Hannum’s, PhenoAge and Skin and Blood clocks are presented in Figure 2 and Figure S1 (Supplementary Material). The correlation coefficients between CA and EA were between 0.688, *p* < 0.001 (for PhenoAge) and 0.856, *p* < 0.001 (for Skin and Blood age), Figure S2 (Supplementary Material). Sex-specific distribution of the epigenetic age acceleration for all four clocks is shown on Supplementary Figure S3.

The mean ΔAHr, ΔAHn and ΔASB were significantly lower in MI/ACS cases compared to controls, 0.055 (5.35) vs. 1.66 (5.09), *p* = 0.008, −2.70 (5.36) vs. −1.16 (4.82), *p* = 0.009 and −2.55 (4.06) vs. −1.55 (3.58), *p* = 0.023, correspondingly (Table 1). ΔAPh was similar in cases and controls. Sex-specific distribution of the epigenetic age acceleration for all four clocks is shown on Supplementary Figure S3.

### 3.2. Association between Age Acceleration and Risk of MI/ACS

Odds ratios of MI/ACS per 1–year increment of EA measures, modeled as a continuous variable, are presented in Table 2. ORs of MI/ACS per 1–year increment of EA measures were 1.016 (95% CI 0.96–1.07) for ΔAHr; 1.023 (95% CI 0.95–1.08) for ΔAHn; 1.032 (95% CI 0.99–1.07) for ΔAPh; and 1.002 (95% CI 0.94–1.07) for ΔASB in age-adjusted models (Table 2). In multivariable analyses (fully adjusted Model 4), the ORs were 1.009 (95% CI 0.95–1.07), 1.012 (95% CI 0.95–1.08), 1.017 (95% CI 0.97–1.06) and 1.009 (95% CI 0.93–1.09), respectively, and were not statistically significant. 

The results are presented separately for men and women in Table S1 and Figure S4 (Supplementary materials). The relationships between EAA and MI/ACS were of the same directions compared to pooled results and were not statistically significant.

Odds ratios of MI/ACS by tertiles of EA measures are presented in Table 3. After controlling for age, the risk of MI/ACS was modestly higher in ΔAHr tertile 3 vs. tertile 1: OR = 1.26 (95% CI 0.65–2.44). Similarly, the risk of MI/ACS was higher in tertile 3 of ΔAHn compared with the lowest tertile, the OR was 1.57 (95% CI 0.79–3.14). In multivariable models adjusted for age, sex, smoking, SBP, BMI, total cholesterol and education, the ORs were 1.24 (95% CI 0.60–2.56) and 1.36 (95% CI 0.63–2.96), respectively. However, as the lower margin of 95% confidence intervals was always less than 1.00, it is possible that these results arose by chance alone.

The risk of MI/ACS increased in tertile 3 vs. tertile 1 of ΔAPh, with OR = 2.09 (95% CI 1.11–3.94), *p* = 0.022 independent of age, and a statistically not significant OR of 1.8 (CI 95% 0.99-3.52), *p* = 0.065 was found in the sex- and age-adjusted Model 2 (Table 2). This association was partly explained (or mediated) by smoking and metabolic factors (blood pressure, body mass index, total and LDL cholesterol). The relationships between tertiles of ΔAHn and MI/ACS were also positive but statistically not significant. The second tertile of ΔAHr was negatively related to MI/ACS in any type of adjustment. There was no association found between tertiles of baseline ΔASB and the risk of MI/ACS. 

For internal validation, we also assessed the association between MI/ACS and EA measures in case and expanded control groups (Tables S2–S4). The results were similar but somewhat weaker for continuous epigenetic age acceleration (EAA) and for EAA by tertiles than in the original case-control groups. In the expanded sample, the age-adjusted OR of MI/ACS per 1–year increment of EAA was 1.014 (95% CI 0.98–1.05) for ΔAPh. The risk of MI/ACS was higher in the top tertile 3 vs. tertile 1 for ΔAPh, with OR = 1.42 (95% CI 0.85–2.42), but statistically not significant in the age-adjusted model and with further adjustment. For ΔAHn, the relationships with MI/ACS were of a similar direction but weaker still; ΔAHr and ΔASB were not associated with the risk of MI/ACS.

## 4. Discussion

In this nested case-control study in Novosibirsk (Russia), we selected CVD-free participants with incident MI/ACS (cases) and age- and sex-frequency matched controls from a population-based cohort (HAPIEE); participants were followed up over 15 years. Epigenetic ages derived from DNAm with Horvath’s, Hannum’s and Skin and Blood clocks were close to the chronological ages, but PhenoAge’s predictions were less close to CA. From the EA indices tested in this study, the relationship between incident MI/ACS and 1–year increments of the difference between baseline EA and CA assessed by the PhenoAge clock was positive but statistically not significant. The relationships between the risk of MI/ACS and acceleration of EA assessed by ΔAHr, ΔAHn and ΔASB were of the same direction but were weaker and also statistically not significant. 

When EAA was classified into tertiles, the risk of MI/ACS modestly increased in tertile 3 vs. tertile 1 of EAA assessed by the PhenoAge clock only in the minimally adjusted model independent of age and was borderline in the age- and sex-adjusted model. This association appeared to be explained (or mediated) by smoking and metabolic factors. We did not find significant associations between EAA tertiles of other studied DNAm clocks and MI/ACS in our sample.

Age is one of the strongest risk factors for many human diseases, including CVD and, specifically, CHD [16,24]. Given the significance of biological aging, a variety of estimators of biological age were constructed. DNAm-based estimators (epigenetic age) precisely predict chronological age and their positive deviation from chronological age is considered as ‘accelerated biological aging’ (EAA). 

In our dataset, EAA was higher in the control group than in MI/ACS cases. That could be explained by the differences in chronological ages in case and control group subjects. Indeed, it is known that EAA is non-linear and tends to decrease with age [10], and in our sample, the chronological age at the time of blood draw in the control subjects is lower than that of the cases. To address the potential interaction between chronological age at baseline and EAA, an analysis stratified by age group would be needed; unfortunately, our study was not large enough to do so. 

Evidence is growing on the association between epigenetic age and risk of all-cause mortality and some cause-specific mortality [13,19,20,21,22,23,24]. In a recent meta-analysis, Fransquet et al. (2019) (41,607 subjects) defined that each 5–year increase in epigenetic age acceleration (EAA) was associated with 8% and 15% increased risk of all-cause mortality (by Hannum’s and Horvath’s, correspondingly) [24]. Another meta-analysis of Marioni et al. (2015) [22], based on four large cohorts (two Lothian Birth Cohorts, Framingham Heart Study and Normative Aging Study), revealed a pooled effect of 16% and 9% increases in total mortality risk by 5–year higher EAA (by Hannum’s and Horvath’s, respectively).

EAA has been extensively investigated in relation to age-dependent diseases, health, lifestyle and environmental factors, with inconsistent results [15,21,25,39,40,41,42]. In our study, the relationship between MI/ACS and PhenoAge acceleration by 1–year was positive but statistically not significant. We found modestly increased risk of incident MI/ACS in the top vs. the lowest tertile of baseline difference between EA and CA confined to the PhenoAge clock in the minimally adjusted model. The direction of association for PhenoAge is broadly in line with associations between EA acceleration and MI or CHD risk reported in a meta-analysis of five cohorts [13], in the NAS and KORA F4 cohorts [43] and in comparative analysis between GrimAge and other EA estimators [17]. For instance, in the meta-analysis of the NAS cohort (*n* = 737 white men) and KORA F4 cohort (*n* = 1725) with follow-up ranging from 8.5–14 years, the HR of MI was 1.15 per 1 SD of PhenoAge acceleration [43]. In a meta-analysis of five cohorts (WHI (two cohorts), FHS, NAS, JHS), a 1–year increase in PhenoAge was associated with CHD risk with β=0.016 to 0.073 [13]. A recent systematic review based on 156 publications and a meta-analysis of 57 factors by Oblack et al. [15] obtained similar effects, with HR for CVD risk ranging from 1.011 to 1.083 per year assessed by four epigenetic clocks (Horvath’s, Hannum’s, PhenoAge and GrimAge).

We observed ORs of MI/ACS ranging from 1.009–1.012 per 1 year of EAA and from 1.2–1.3 in the top tertile vs. the lowest tertile of EAA by Horvath’s and Hannum’s clocks; these coefficients were not statistically significant but close to those previously reported in the ARIC study [44], a German ESTHER case cohort [20] and cumulative data from a recent meta-analysis [15]. For example, in the ARIC study of a sub-cohort of black participants (*n* = 2543) followed for 21 years, the HR of fatal CHD was 1.17 and 1.22 per 5–year increment of Hannum’s and Horvath’s EAA independent of other factors; the HR for MI modestly increased by 1.12 for Hannum’s EAA [44]. In the ESTHER case cohort study (*n* = 1864), the HR of CVD mortality was 1.19 for a 5–year increment of Horvath’s EAA independent of other factors and similar but weaker for Hannum’s EAA measure [20]. On the other hand, for example, Horvath et al. (2016) [25] did not report an association between EAA and incident CHD in the Women’s Health Initiative dataset.

The OR in our analysis for EAA by PhenoAge tertile was substantially attenuated after controlling for metabolic factors (BMI, SBP, TC) and education. The residual associations, although still of meaningful magnitude, had wide confidence intervals, and they may have arisen by chance alone on account of our relatively small sample size. This is in contrast to some of the larger aforementioned studies that have reported associations in multivariable-adjusted models. 

The impact of metabolic risk factors to mediate the relationship between epigenetic age and risk of atherosclerotic CVD and acute coronary outcomes is well supported. Original studies and comprehensive reviews consistently demonstrate that BMI is strongly correlated with epigenetic age, independent of other covariates [15,21,39,45]. Obesity is a known risk factor for many age-related diseases; it is associated with oxidative stress and a pro-inflammatory state that enhances white blood cell turnover and is considered as pro-aging [39]. The exact mechanisms linking DNA methylation profiles and CVD are not entirely clear, but DNAm is considered as a key player in the genetic regulation of genes related to cardiac homeostasis [46]. In the last decade, several DNAm studies (including EWASs) have linked CHD and atherosclerosis to differentially methylated sites related to genes most commonly involved to the pathways of obesity, adiposity, lipid and carbohydrate metabolism, inflammation, macrophage activity, smooth muscle cell proliferation and renin–angiotensin regulation [45,47,48,49,50,51].

The diversity across previous studies in the relationship between EA and CVD/CHD regarding the presence and magnitude of association might be related to the heterogeneity of the studied outcomes and study design, age, ethnic and sex composition and volume of the sample, population-specific characteristics of morbidity, risk factor profiles and environmental exposure, covariates and multiple statistical testing, as well as the exact DNAm platform and EA clocks used in the analysis.

## 5. Study Limitations and Advantages

The study has several limitations, particularly the relatively small sample size. In this nested case-control study, we randomly selected cases of incident fatal and non-fatal MI and ACS among all new-onset CHD events occurring in a large population cohort (9360) during a 15-year follow-up; the cases and controls were frequency matched by age and sex. This makes it more likely that we obtained a representative sample of typical acute coronary disease for this population, but the study was under-powered to study relatively small effects. The sample size was determined by the numbers of eligible events with DNA samples and by the cost of the lab analyses. In post hoc power calculations (with 160 cases and 240 controls), the estimated statistical power of the analysis to detect a difference between cases and controls in delta (epigenetic age–chronological age) of 1.5 and 2 years was 80% > 90%, respectively. For internal validation, we repeated the analysis using another (expanded) control group which did not significantly alter the results. However, further enlarging of the MI/ACS sample would certainly improve the statistical power to detect significant associations for EAA metrics.

For practical reasons, we used frequency matching of cases and controls by 5–year age groups and sex instead of an individual matching procedure. After exclusions by quality control, the distribution of age groups between cases and controls slightly changed but the statistical adjustment would take this into account. Sex distribution remained practically uniform (50–60%) between cases and controls. We also conducted internal validation using an expanded control sample with age–sex distribution closer to cases (mean age 59.8 years in cases vs. 57.5 years in expanded controls); the use of an expanded sample did not significantly alter the results.

To protect against misclassification which could not be excluded for CHD as a potentially heterogonous outcome, we focused on the most strictly defined categories of CHD (MI and ACS) ascertained from the data of ‘MI Register’ using standardized and internationally validated criteria. To ensure completeness of registration, we additionally used overlapping sources of case ascertainment and both hot-pursuit and cold-pursuit approaches were combined by MI Register.

Another potential limitation relates to the arguable differences in DNAm between sexes [50]. To overcome this limitation, the sampling procedure kept the sex distribution uniform among cases and controls (nearly 50–60%), and we adjusted the estimates by sex (Model 2). Finally, in the sensitivity analysis split by sex we received ORs of the same directions and similar values compared to pooled results and they were insignificant (data are shown in Supplement Material, Table S1).

Our study also has several strengths. First, it is the first population-based prospective nested case-control study exploring the relationship between epigenetic age and risk of incident MI/ACS in the Russian population as well as in the Eastern European population. 

Second, we used the latest platform, Illumina Epic 850 BeadChip, for DNA methylation analysis, applied standardized multistep quality control and included longitudinal analysis at a different time point. We only used high-quality data from samples with less than 1% CpGs with detection p-values above the threshold 0.01, and CpGs with bead count numbers of at least 3 and p-values below 0.01 across at least 99% of samples. 

Third, we used four estimators of epigenetic age (Horvath’s, Hannum’s, PhenoAge and Skin and Blood clocks) with established precision in estimating chronological age, age-related diseases and mortality [16] and constructed with a variety of approaches (blood-based, pan-tissue or phenotype-based). 

Finally, our data provide the first evidence of the magnitude and potential consequences of EAA in the Russian population. 

## 6. Conclusions

In this case-control study nested in a prospective population-based cohort, we did not find strong associations between accelerated epigenetic age markers and risk of MI/ACS. There was a modest association between acceleration of epigenetic age and increased risk of MI/ACS confined only to the highest tertile of the PhenoAge clock, which appeared to be partly modulated by smoking and metabolic factors. However, this isolated positive finding may have been a false positive result and needs to be interpreted with caution. If confirmed in larger studies, however, epigenetic age acceleration may prove to be a useful predictor of the risk of acute coronary events in older age, with a potential for practical implications for CVD prevention.

## Figures and Tables

**Table 1:** Distribution of baseline covariates among cases of incident MI/ACS and control (the Russian arm of the HAPIEE study).

Covariates	Cases (Incident MI/ACS)	Controls	*p*-Value ^a^
Observed	129	177	
Age at baseline, years (mean, SD)	59.8 (6.87)	54.5 (6.45)	<0.001
Females (%)	62 (48.1)	73 (58.8)	0.064
Systolic blood pressure, mmHg (mean, SD)	151.6 (26.93)	133.2 (21.87)	<0.001
Diastolic blood pressure, mmHg (mean, SD)	92.3 (14.36)	86.0 (12.69)	<0.001
Body mass index, kg/sqm (mean, SD)	28.8 (5.73)	27.50 (4.90)	0.031
Waist/hip ratio, unit (mean, SD)	0.90 (0.077)	0.87 (0.087)	0.002
Total cholesterol mmol/L (mean, SD)	6.61 (1.27)	6.42 (1.28)	0.204
LDL cholesterol, mmol/L (mean, SD)	4.32 (1.14)	4.15 (1.13)	0.207
Glucose, plasma, mmol/L mean, SD)	6.41 (2.29)	5.77 (0.85)	0.001
Hypertension (%)	96 (74.4)	80 (45.2)	<0.001
HT treatment (among HT), (%)	46 (47.9)	46 (27.5)	0.006
Diabetes mellitus type 2 (%)	24 (18.9)	10 (5.8)	<0.001
DM2 treatment (among DM2), (%)	8 (33.3)	3.(30.0)	0.850
Frequency of drinking (%) Non-drinkers	24 (18.6)	15 (8.5)	0.050
<1/month	55 (42.6)	76 (42.9)	
1–3/month	25 (19.4)	35 (19.8)	
1–4/week	22 (17.1)	48 (27.1)	
5+/week	3 (2.3)	3 (1.7)	
Smoking (%) Never smoked	75 (58.1)	105 (59.3)	0.066
Former smoking	10 (7.8)	27 (15.3)	
Present smoker	44 (34.1)	56 (31.6)	
Married (%)	96 (74.4)	135 (76.3)	0.405
University education (%)	27 (20.9)	56 (31.6)	<0.001
Difference EA–chronological age by four measures:			
ΔAHr, year	0.055 (5.35)	1.663 (5.09)	0.008
ΔAHn, year	−2.702 (5.36)	−1.161 (4.82)	0.009
ΔAPh, year	−8.945 (6.43)	−8.762 (6.38)	0.806
ΔASB, year	−2.551 (4.06)	−1.550 (3.58)	0.023

SD—standard deviation; EA—epigenetic age; CVD—cardiovascular disease. ^a^—ANOVA or chi-square test.

**Table 2:** Relationship between MI/ACS and epigenetic age acceleration, per 1–year increment of the difference between baseline EA and CA (cases, *n* = 129 and controls, *n* = 177).

Measure of Epigenetic Age	*n*, Case/Control	Model 1	Model 2	Model 3	Model 4
		OR (95% CI)	OR (95% CI)	OR (95% CI)	OR (95% CI)
ΔAHr, per 1 year	129/177	1.016 (0.96–1.07)	1.003 (0.87–1.36)	1.008 (0.95–1.06)	1.009 (0.95–1.07)
*p*-value for trends		0.563	0.911	0.785	0.763
ΔAHn, per 1 year	129/177	1.023 (0.95–1.08)	1.001 (0.95–1.06)	1.006 (0.95–1.07)	1.012 (0.95–1.08)
*p*-value for trends		0.418	0.961	0.842	0.708
ΔAPh, per 1 year	129/177	1.032 (0.99–1.07)	1.021 (0.98–1.06)	1.017 (0.98–1.06)	1.017 (0.97–1.06)
*p*-value for trends		0.126	0.310	0.430	0.459
ΔASB, per 1 year	129/177	1.002 (0.94–1.07)	0.991 (0.93–1.06)	0.997 (0.93–1.07)	1.009 (0.93–1.09)
*p*-value for trends		0.962	0.802	0.927	0.825

ΔAHr—difference between EA by Horvath’s and chronological age; ΔAHn—difference between EA by Hannum’s and chronological age; ΔAPh—difference between phenotypic EA and chronological age; ΔASB—difference between Skin and Blood EA and chronological age; OR—odds ratio; CI—confidence interval; Model 1: age-adjusted; Model 2: adjusted for age and sex; Model 3: adjusted for age, sex and smoking; Model 4: adjusted for age, sex, smoking, SBP, TC, BMI and education.

**Table 3:** Relationship between MI/ACS and epigenetic age acceleration by tertiles of the difference between baseline EA and CA (cases, *n* = 129 and controls, *n* = 177).

Measure of Epigenetic Age	*n*, Case/Control	Tertiles	Absolute Difference T1-T2 T2-T3	Model 1	Model 2	Model 3	Model 4
				OR (95% CI)	OR (95% CI)	OR (95% CI)	OR (95% CI)
ΔAHr, year	129/177	T1 (ref)		1.0	1.0	1.0	1.0
		T2	5.64	0.89 (0.49–1.63)	0.83 (0.45–1.53)	0.83 (0.44–1.54)	0.91 (0.47–1.77)
		T3	5.48	1.26 (0.65–2.44)	1.14 (0.59–2.22)	1.21 (0.61–2.40)	1.24 (0.60–2.56)
		*p*-value for trends		0.510	0.738	0.624	0.593
ΔAHn, year	129/177	T1 (ref)		1.0	1.0	1.0	1.0
		T2	5.35	1.28 (0.68–2.39)	1.20 (0.63–2.24)	1.26 (0.66–2.40)	1.22 (0.61–2.44)
		T3	5.40	1.57 (0.79–3.14)	1.26 (0.61–2.60)	1.36 (0.65–2.85)	1.36 (0.63–2.96)
		*p*-value for trends		0.198	0.526	0.408	0.437
ΔAPh, year	129/177	T1 (ref)		1.0	1.0	1.0	1.0
		T2	6.49	1.19 (0.64–2.21)	1.18 (0.63–2.20)	1.21 (0.65–2.28)	1.17 (0.61–2.27)
		T3	7.40	2.09 (1.11–3.94)	1.84 (0.99–3.52)	1.78 (0.92–3.43)	1.64 (0.82–3.31)
		*p*-value for trends		0.022	0.065	0.088	0.171
ΔASB, year	129/177	T1 (ref)		1.0	1.0	1.0	1.0
		T2	3.94	0.88 (0.47–1.62)	0.80 (0.43–1.51)	0.84 (0.45–1.58)	0.99 (0.50–1.94)
		T3	4.06	1.13 (0.60–2.11)	1.00 (0.53–1.89)	1.09 (0.57–2.09)	1.18 (0.60–2.37)
		*p*-value for trends		0.699	0.948	0.738	0.637

ΔAHr—difference between EA by Horvath’s and chronological age; ΔAHn—difference between EA by Hannum’s and chronological age; ΔAPh—difference between phenotypic EA and chronological age; ΔASB—difference between Skin and Blood EA and chronological age; OR—odds ratio; CI—confidence interval; Model 1: age-adjusted; Model 2: adjusted for age and sex; Model 3: adjusted for age, sex and smoking; Model 4: adjusted for age, sex, smoking, SBP, TC, BMI and education.

## Data Availability

The data presented in this study are available in tabulated form on request. The data are not publicly available due to ethical restrictions and project regulations.

## Supplementary Materials

The following are available online at https://www.mdpi.com/article/10.3390/jpm12010110/s1, Figure S1: Boxplots showing age distribution in case and control groups for A—all samples, B—males and C—females. Figure S2: Scatterplots of chronological vs. epigenetic age by Horvath’s, Hannum’s, PhenoAge and Skin and Blood clocks. Black line corresponds to the predicted age equal to the chronological age. Figure S3: Correlation chart for the chronological age and four DNAm age predictions. The panels on the main diagonal show distributions of the CA and DNAm clock results, the panels below the diagonal are scatter plots with a fitted line, the panels above the diagonal show the corresponding Pearson correlation coefficients with significance level below 0.001. Figure S4: Density plots and boxplots of the age acceleration distribution for (A) Horvath’s, (B) Hannum’s, (C) PhenoAge, (D) Skin and Blood epigenetic clocks for sex- and case-control-specific groups. The numbers on the right side of the figure corresponds to *t*-test *p*-values. Table S1: Relationship between MI/ACS and epigenetic age acceleration, per 1–year increment of the difference between baseline EA minus CA in men and women (men, *n* = 171, women, *n* = 135). Table S2: Distribution of baseline covariates among cases of incident MI/ACS and expanded control sample (cases, *n* = 129 and controls, *n* = 265). Table S3: Relationship between MI/ACS and epigenetic age acceleration, per 1–year increment of the difference between baseline EA and CA in the expanded sample (cases, *n* = 129 and controls, *n* = 265). Table S4: Relationship between MI/ACS and epigenetic age acceleration in the expanded sample, by tertiles of the difference between baseline EA and CA (cases, *n* = 129 and controls, *n* = 265).
