# Supplementary material for: The Relationship between Epigenetic Age and Myocardial Infarction/Acute Coronary Syndrome in a Population-Based Nested Case-Control Study
Source: J Pers Med. 2022 Jan 14;12(1):110. doi: 10.3390/jpm12010110 (PMC8781885; doi:10.3390/jpm12010110)
Supplement: Supplementary file 1 [file jpm-12-00110-s001.zip › Supplementary caption_corrected.pdf]

Journal of Personalized Medicine

Supplementary Materials: The following are available online at www.mdpi.com/xxx/s1,

Figure S1: Boxplots showing age distribution in case and control groups for A - all samples, B - Males and C - females.

Figure S2: Scatterplots of chronological vs epigenetic age by Horvath, Hannum, PhenoAge and Skin and Blood clocks. Black line corresponds to the Predicted age equal to the Chronological age.

Figure S3: Correlation chart for the chronological age and four DNAm age predictions. The panels on the main diagonal show distributions of the CA and DNAm clock results, the panels below the diagonal are scatter plots with a fitted line, the panels above the diagonal show the corresponding Pearson correlation coefficients with significance level below 0.001).

Figure S4: Density plots and boxplots of the age acceleration distribution for **(A)** Horvath, **(B)** Hannum, **(C)** PhenoAge, **(D)** Skin and Blood epigenetic clocks for sex- and case-control specific groups. The numbers on the right side of the Figure correspond to t-test p-values.

Table S1: Relationship between MI/ACS and epigenetic age acceleration, per 1 year increment of the difference between baseline EA minus CA in men and women (men n =171, women, n = 135).

Table S2: Distribution of baseline covariates among cases of incident MI/ACS and expanded control (cases, n = 129 and controls, n =265).

Table S3: Relationship between MI/ACS and epigenetic age acceleration, per 1 year increment of the difference between baseline EA and CA in the expanded sample (cases, n = 129 and controls, n =265).

Table S4: Relationship between MI/ACS and epigenetic age acceleration in the expanded sample, by tertiles of the difference between baseline EA minus CA (cases, n=129 and controls, n=265).
