# Supplementary material for: The Relationship between Epigenetic Age and Myocardial Infarction/Acute Coronary Syndrome in a Population-Based Nested Case-Control Study
Source: J Pers Med. 2022 Jan 14;12(1):110. doi: 10.3390/jpm12010110 (PMC8781885; doi:10.3390/jpm12010110)
Supplement: Supplementary file 1 [file jpm-12-00110-s001.zip › Supplement_Tables_REVISION_corrected.pdf]

Journal of Personalized Medicine

Table S1. Relationship between MI/ACS and epigenetic age acceleration, per 1 year increment of the difference between baseline EA minus CA in men and women (men n =171, women, n = 135).

Measure of epigenetic age	n, case/ control	Model1	Model2	Model3
		OR (95%CI)	OR (95%CI)	OR (95%CI)
**Men**				
ΔAHr, per 1 year	67/104	1.000 (0.93-1.08)	1.006 (0.93-1.08)	1.000 (0.92-1.08)
	p-value for trends	*0.994*	*0.880*	*0.993*
ΔAHn, per 1 year	67/104	0.995 (0.92-1.08)	1.000 (0.92-1.09)	1.000 (0.91-1.09)
	p-value for trends	*0.903*	*0.992*	*0.991*
ΔAPh, per 1 year	67/104	1.035 (0.98-1.09)	1.026 (0.971.09)	1.026 (0.96-1.09)
	p-value for trends	*0.239*	*0.401*	*0.426*
ΔASB, per 1 year	67/104	0.986 (0.89-1.09)	0.989 (0.89-1.09)	0.988 (0.89-1.10)
	p-value for trends	*0.782*	*0.831*	*0.825*
**Women**				
ΔAHr, per 1 year	62/73	1.008 (0.93-1.09)	1.013 (0.93-1.10)	1.037 (0.95-1.14)
	p-value for trends	*0.994*	*0.763*	*0.435*
ΔAHn, per 1 year	62/73	1.009 (0.93-1.10)	1.016 (0.94-1.10)	1.033 (0.94-1.13)
	p-value for trends	*0.824*	*0.708*	*0.492*
ΔAPh, per 1 year	62/73	1.008 (0.95-1.07)	1.004 (0.95-1.06)	1.008 (0.94-1.8
	p-value for trends	*0.788*	*0.904*	*0.806*
ΔASB, per 1 year	62/73	0.999 (0.91-1.10)	1.005 (0.91-1.8)	1.031 (0.92-1.15)
	p-value for trends	*0.980*	*0.926*	*0.592*

ΔAHr – difference between EA by Horvath and chronological age; ΔAHn – difference between EA by Hannum and chronological age; ΔAPh – difference between Phenotypic EA and chronological age; ΔASB – difference between Skin and Blood EA and chronological age; OR – odds ratio ratio; CI – confidence interval; Model 1: age-adjusted; Model 2: adjusted for age and sex; Model 2:– adjusted for age, and smoking; model 3: adjusted for age, smoking, SBP, TC, BMI and education.

Table S2. Distribution of baseline covariates among cases of incident MI/ACS and expanded control(cases, n = 129 and controls, n =265).

Covariates	Cases (incident MI/ACS)	Expanded controls	p-value
Observed	129	265	
Age at baseline, years (mean, SD)	59.8 (6.87)	57.4 (6.95)	<0.001
Females (%)	62 (48.1)	155 (58.5)	0.051
Systolic blood pressure, mmHg (mean, SD)	151.6 (26.93)	136.7 (23.10)	<0.001
Diastolic blood pressure, mmHg (mean, SD)	92.3 (14.36)	86.5 (12.55)	<0.001
Body mass index, kg/sqm (mean, SD)	28.8 (5.73)	27.83 (4.83)	0.073
Waist/hip ratio, unit (mean, SD)	0.90 (0.077)	0.88 (0.032)	0.006
Total cholesterol mmol/l (mean, SD)	6.61 (1.27)	6.44 (1.26)	0.210
LDL cholesterol, mmol/l (mean, SD)	4.32 (1.14)	4.19 (1.12)	0.287
Glucose, plasma, mmol/l mean, SD)	6.41 (2.29)	5.82 (0.92)	<0.001
Hypertension (%)	96(74.4)	136 (51.3)	<0.001
HT treatment (among HT), (%)	46 (47.9)	55 (40.4)	0.001
Diabetes mellitus type 2 (%)	24(18.9)	16 (6.3)	<0.001
DM2 treatment (among DM2), (%)	8 (33.3)	5.(31.3)	0.840
Frequency of drinking (%)			
Non-drinkers	24 (18.6)	28 (10.6)	0.125
< 1/month	55 (42.6)	116 (43.8)	
1-3/month	25 (19.4)	50 (18.9)	
1-4/week	22 (17.1)	67 (25.3)	
5+/week	3 (2.3)	4 (1.5)	
Smoking (%)			
Never smoking	75 (58.1)	163 (61.5)	0.006
Former smoking	10 (7.8)	44 (16.6)	
Present smoker	27 (20.9)	58 (21.9)	
Married (%)	96 (74.4)	192 (72.5)	0.680
University education (%)	27 (20.9)	89 (33.6)	<0.001
Difference EA-chronological age by four measures:			
ΔAHr, year	0.055 (5.35)	1.213 (4.92)	0.034
ΔAHn, year	-2.702 (5.36)	-1. 831 (4.64)	0.098
ΔAPh, year	-8.945 (6.43)	-8.977 (6.01)	0.960
ΔASB, year	-2.551 (4.06)	-1.551 (3.50)	0.012

SD – standard deviation; EA- epigenetic age; CVD – cardiovascular disease
[a] ANOVA or Chi-square test.

Table S3. Relationship between MI/ACS and epigenetic age acceleration, per 1 year increment of the difference between baseline EA minus CA in the expanded sample (cases, n = 129 and controls, n =265).

Measure of epigenetic age	n, case/ control	Model1	Model2	Model3	Model4
		OR (95%CI)	OR (95%CI)	OR (95%CI)	OR (95%CI)
ΔAHr, per 1 year	129/177	0.98 (0.94-1.03)	0.969 (0.92-1.06)	0.971 (0.93-1.02)	0.971 (0.92-1.02)
	p-value for trends	*0.391*	*0.193*	*0.230*	*0.258*
ΔAHn, per 1 year	129/177	0.996 (0.95-1.05)	0.979 (0.93-1.03)	0.984 (0.93-1.04)	0.995 (0.94-1.05)
	p-value for trends	*0.886*	*0.413*	*0.554*	*0.860*
ΔAPh, per 1 year	129/177	1.014 (0.98-1.05)	1.005 (0.97-1.04)	1.000 (0.961.04)	0.998 (0.96-1.04)
	p-value for trends	*0.439*	*0.809*	*0.988*	*0.923*
ΔASB, per 1 year	129/177	0.950 (0.89-1.01)	0.938 (0.88-1.00)	0.947 (0.89-1.1	0.949 (0.88-1.02)
	p-value for trends	*0.096*	*0.055*	*0.088*	*0.135*

ΔAHr – difference between EA by Horvath and chronological age; ΔAHn – difference between EA by Hannum and chronological age; ΔAPh – difference between Phenotypic EA and chronological age; ΔASB – difference between Skin and Blood EA and chronological age; OR – odds ratio; CI – confidence interval;
Model 1: age-adjusted; Model 2: adjusted for age and sex; Model 3: adjusted for age, sex and smoking; Model 4: adjusted for age, sex, smoking, SBP, TC, BMI and education.

Table S4. Relationship between MI/ACS and epigenetic age acceleration in the expanded sample, by tertiles of the difference between baseline EA minus CA (cases, n=129 and controls, n=265).

Measure of epigenetic age	n, case/ control	Tertiles	Absolute difference T1-T2 T2-T3	Model1 OR (95%CI)	Model2 OR (95%CI)	Model3 OR (95%CI)	Model4 OR (95%CI)
ΔAHr, year	129/265	T1 (ref)		1.0	1.0	1.0	1.0
		T2	5.31	0.68 (0.40-1.15)	0.64 (0.38-1.09)	0.61 (0.36-1.05)	0.66 (0.38-1.18)
		T3	5.48	0.86 (0.50-1.49)	0.79 (0.45-1.38)	0.80 (0.45-1.42)	0.83 (0.45-1.51)
		p-value for trend		*0.540*	*0.366*	*0.395*	*0.489*
ΔAHn, year	129/265	T1 (ref)		1.0	1.0	1.0	1.0
		T2	4.94	0.86 (0.50-1.46)	0.79 (0.46-1.36)	0.88 (0.51-1.53)	0.94 (0.52-1.69)
		TQ3	5.31	1.17 (0.65-2.09)	0.97 (0.53-1.78)	1.06 (0.57-1.96)	1.24 (0.64-2.40)
		p-value for trend		*0.626*	*0.894*	*0.888*	*0.550*
ΔAPh, year	129/265	T1 (ref)		1.0	1.0	1.0	1.0
		T2	6.34	0.97 (0.57-1.65)	0.96 (0.56-1.63)	0.98 (0.57-1.68)	0.95 (0.53-1.68)
		T3	7.03	1.42 (0.85-2.42)	1.26 (0.73-2.18)	1.24 (0.70-2.17)	1.10 (0.61-2.00)
		p-value for trend		*0.197*	*0.415*	*0.472*	*0.768*
ΔASB, year	129/265	T1 (ref)		1.0	1.0	1.0	1.0
		T2	3.79	0.54 (0.32-0.93)	0.51 (0.30-0.88)	0.52 (0.29-0.89)	0.56 (0.32-1.00)
		T3	4.05	0.82 (0.48-1.38)	0.73 (0.42-1.25)	0.78 (0.45-1.35)	0.80 (0.44-1.44)
		p-value for trend		*0.432*	*0.241*	*0.363*	*0.424*

ΔAHr – difference between EA by Horvath and chronological age; ΔAHn – difference between EA by Hannum and chronological age; ΔAPh – difference between Phenotypic EA and chronological age; ΔASB – difference between Skin and Blood EA and chronological age; OR – odds ratio; CI – confidence interval;
Model 1: age-adjusted; Model 2: adjusted for age and sex; Model 3: adjusted for age, sex and smoking; Model 4: adjusted for age, sex, smoking, SBP, TC, BMI and education.
