# Supplementary figures and images for: The Relationship between Epigenetic Age and Myocardial Infarction/Acute Coronary Syndrome in a Population-Based Nested Case-Control Study
Source: J Pers Med. 2022 Jan 14;12(1):110. doi: 10.3390/jpm12010110 (PMC8781885; doi:10.3390/jpm12010110)

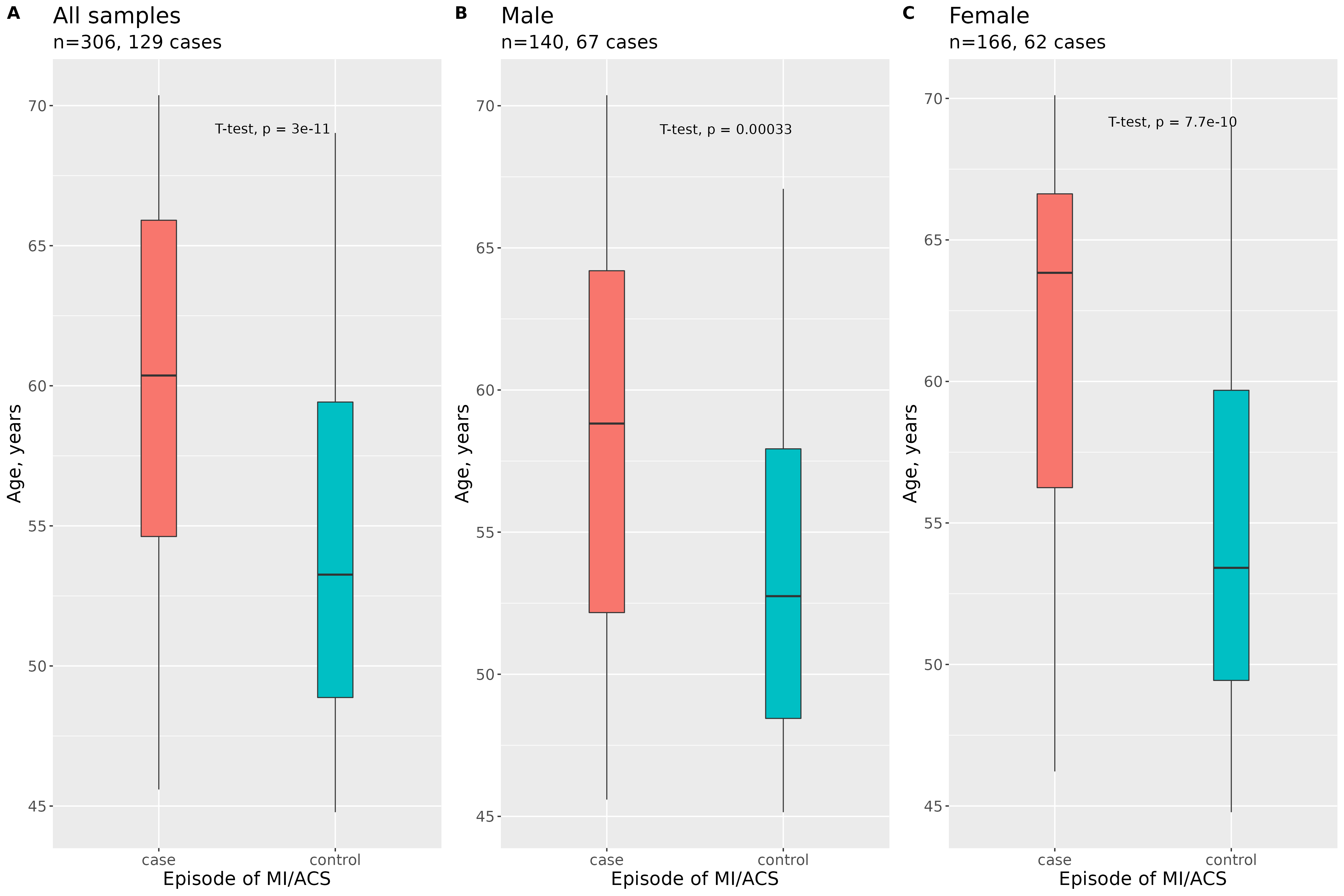

Supplement: Supplementary file 1 [file jpm-12-00110-s001.zip › FigS1_boxplot_age_cc_sex_Revis.png]

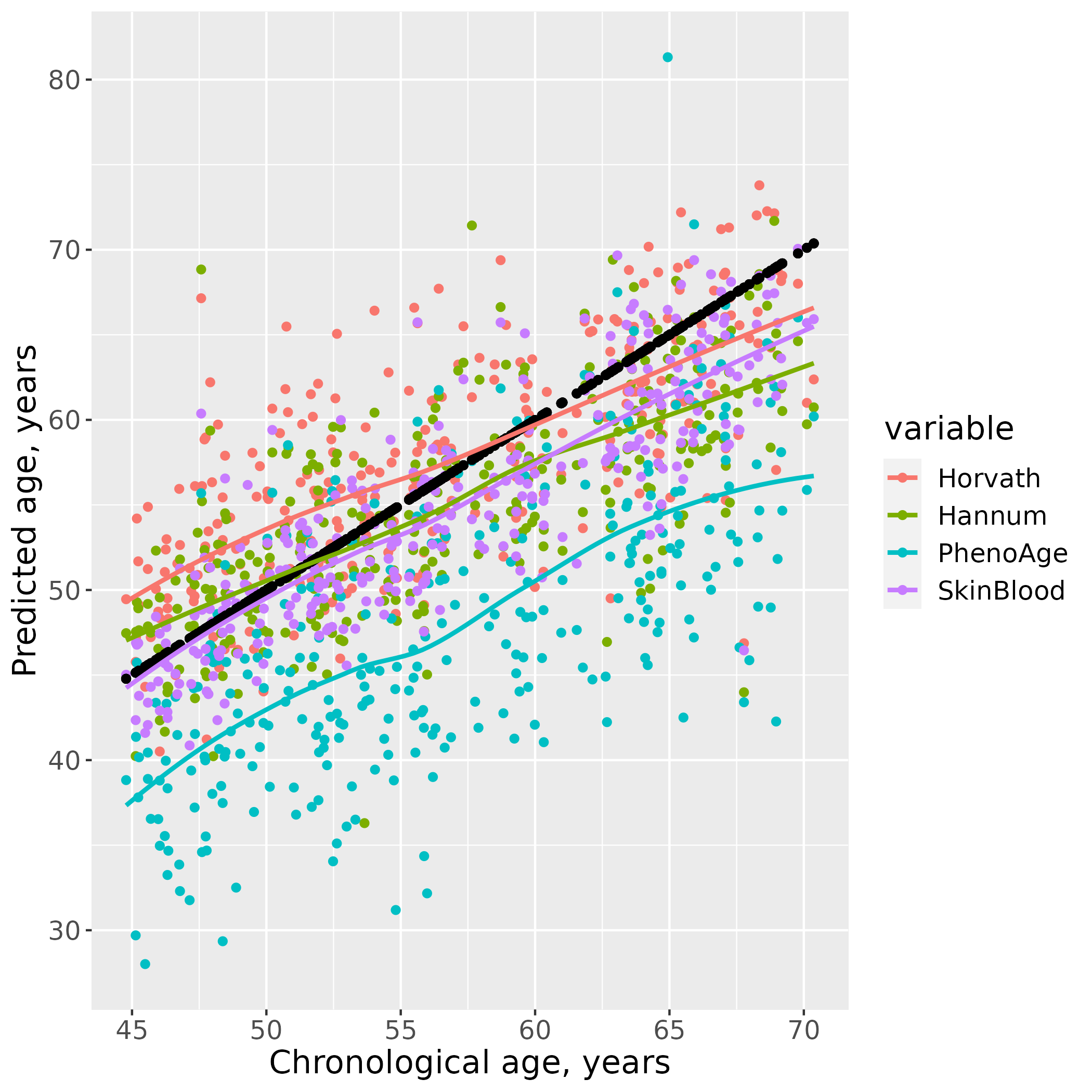

Supplement: Supplementary file 1 [file jpm-12-00110-s001.zip › FigS2_scatter_all_ages_no_pilot_no_title_Revis.png]

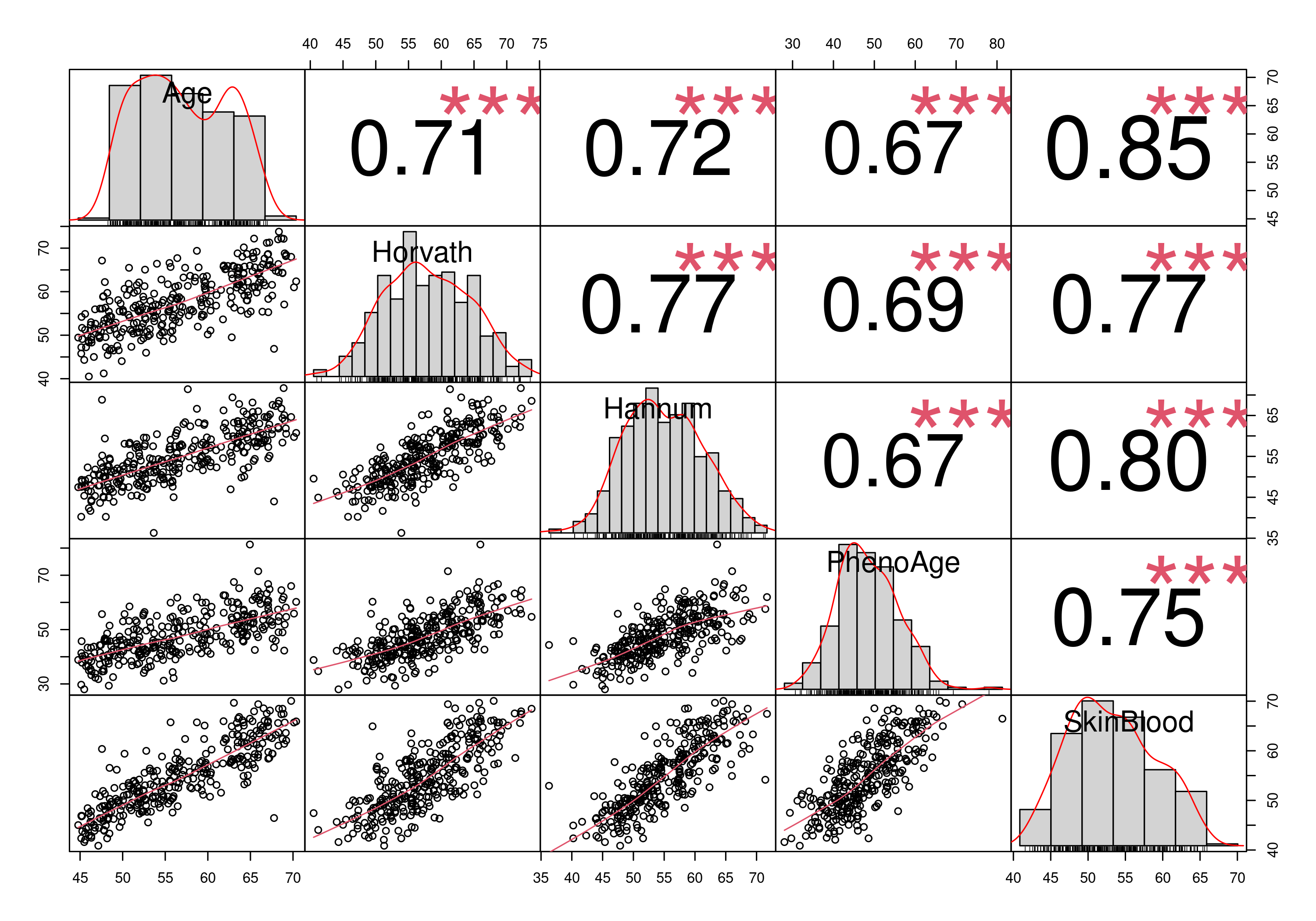

Supplement: Supplementary file 1 [file jpm-12-00110-s001.zip › FigS3_correlation_matrix_epiages_Revis.png]

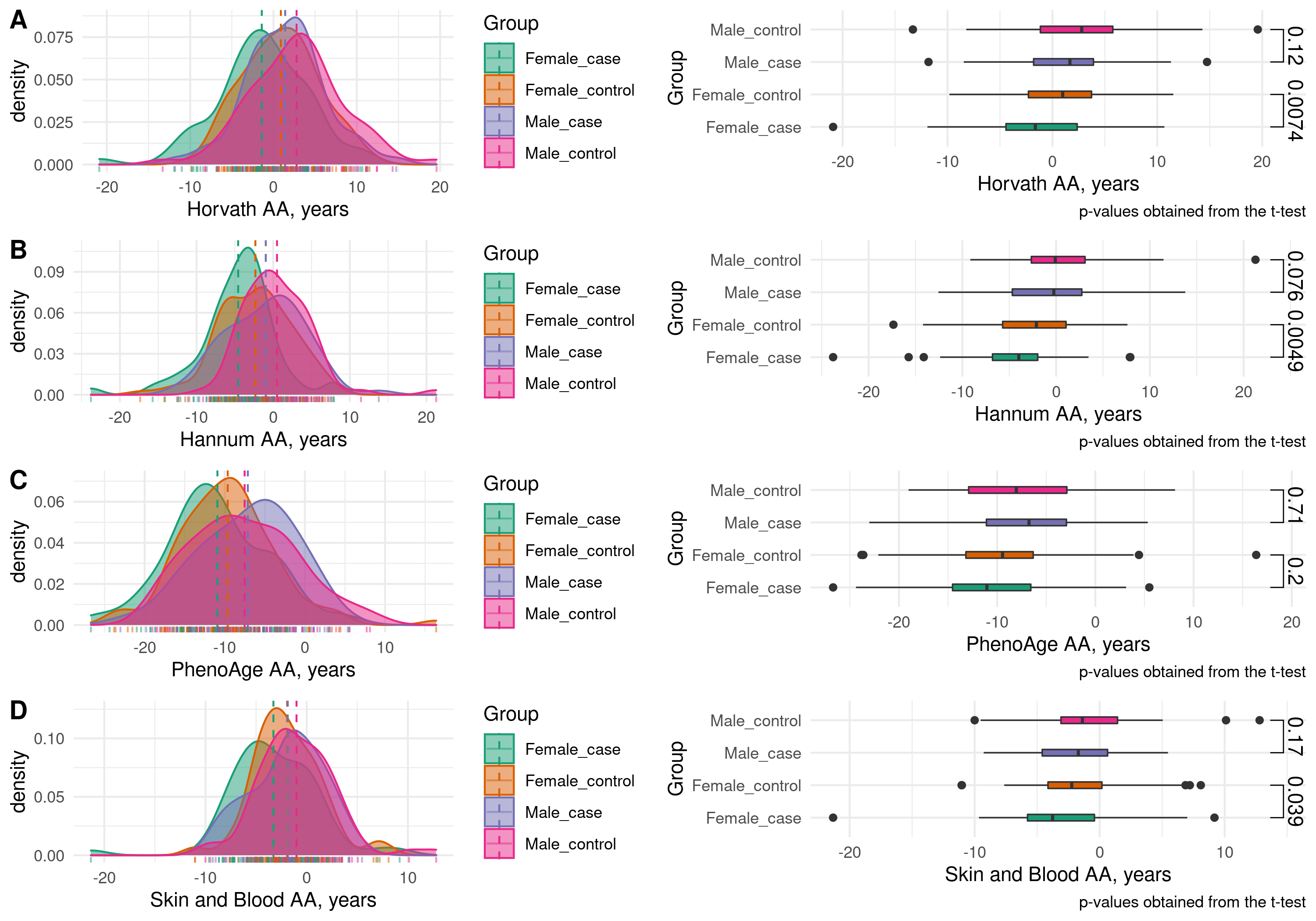

Supplement: Supplementary file 1 [file jpm-12-00110-s001.zip › FigS4_densities_boxplots_sex_cc_aa_Revis.png]
